# Tuning the Structural and Electronic Properties of Zn–Cr LDH/GCN Heterostructure for Enhanced Photodegradation of Estrone in UV and Visible Light

**DOI:** 10.1021/acs.langmuir.4c01897

**Published:** 2024-08-14

**Authors:** Anna Jędras, Jakub Matusik, Esakkinaveen Dhanaraman, Yen-Pei Fu, Grzegorz Cempura

**Affiliations:** † Faculty of Geology, Geophysics and Environmental Protection, Department of Mineralogy, Petrography and Geochemistry, AGH University of Krakow, al. Mickiewicza 30, 30-059 Krakow, Poland; ‡ Department of Materials Science and Engineering, National Dong Hwa University, Shou-Feng, Hualien 97401, Taiwan; § Faculty of Metal Engineering and Industrial Computer Science, International Centre of Electron Microscopy for Materials Science, AGH University of Krakow, al. Mickiewicza 30, 30-059 Krakow, Poland

## Abstract

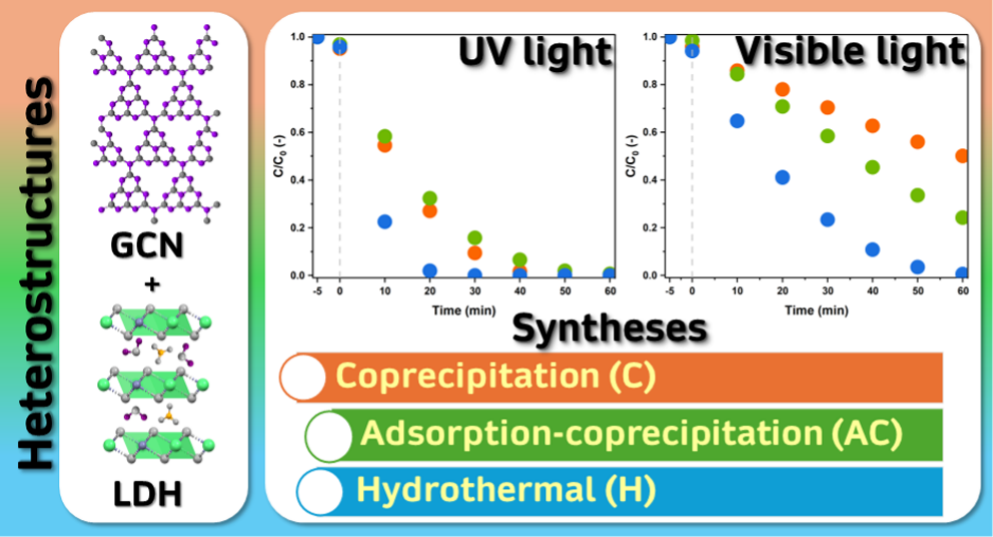

Estrone is an emerging contaminant found in waters and soils all over the world. Conventional water treatment methods are not suitable for estrone removal due to its nonpolarity and low bioavailability. Heterogeneous photocatalysis is a promising approach; however, pristine semiconductors need optimization for efficient estrone photodegradation. Herein, we compared Zn–Cr LDH/GCN heterostructures obtained by three different synthesis methods. The influence of the GCN content in the heterostructure on photoactivity was also tested. The morphology, structure, and electronic properties of the materials were analyzed and compared. The photocatalytic kinetic tests were conducted with 1 ppm of estrone in both UV and visible light, separately. The HLDH-G50 material, obtained by the hydrothermal route and containing 50 wt % of GCN exhibited the highest photocatalytic efficiency. After 1 h, 99.5% of the estrone was degraded in visible light. In UV light, the pollutant concentration was below the detection limit after 0.5 h. The superior effectiveness was caused by numerous factors such as high homogeneity of the formed heterostructure, lower band gap energy of hydrothermal LDH, and increased photocurrent. These characteristics led to prolonged lifetimes of charge carriers, a wider light absorption range, and uniformity of the material for predictable performance. This study highlights the importance of a proper heterostructure engineering strategy for acquiring highly effective photocatalysts designed for water purification. In particular, this work provides innovative insight into comparing different synthesis methods and their influence on materials’ properties.

## Introduction

Endocrine-disrupting compounds (EDCs) are a group of pharmaceuticals with significant endocrine dysfunction effects in humans and animals. These emerging contaminants have neurotoxic, genotoxic, and cancerogenic effects that can vary on the dosage, duration of exposure, and presence of other contaminants.^1,2^ Steroid hormones, both natural and synthetic, are some of the strongest EDCs, even at very low concentrations. Estrogens are steroid hormones excreted by humans and animals in free forms or as conjugates. The latter can be easily biotransformed to the free forms, which are chemically stable.^3^ Estrogens have been detected in waters and soils in many countries. Environmental concentrations in waters may vary between tenths to even thousands of nanograms per liter.^4−6^ Reports state that long exposure to even trace levels of these compounds may contribute to the feminization of the fish in water bodies, low sperm count in adult males, cancers, endometriosis or fibroid, and cardiovascular diseases.^7−9^ It is impossible to fully control the release of estrogenic compounds into the environment, especially natural ones like estrone. Their negative effects must be minimized by water treatment and remediation. Due to the low bioavailability, high persistence, and low concentrations of estrogens, conventional methods of water purification are not efficient in their removal.^3,10^ Physical methods show poor selectivity, clogging issues, secondary pollution (adsorption), high maintenance costs, and membrane fouling (nanofiltration). The biological methods including microbial and enzymatic degradation receive more attention; however, these processes are heavily dependent on several factors: water parameters (temperature and pH) and stability and selectivity of appropriately selected microbes and enzymes.^7^ Removal rate of estrogens during primary treatment is generally low, usually between 10 and 30%, while during secondary treatment with the use of conventional methods, it typically ranges around 40–80%.^3,11,12^

One of the alternative methods of water purification among chemical approaches is heterogeneous photocatalysis, which eliminates pollutants without the production of waste products.^7^ Most commonly it utilizes UV light excitation; however, due to sustainability and cost-efficiency, the use of visible light gains much more interest.^13^ Graphitic carbon nitride (GCN) is a photocatalyst active in both UV and visible light, characterized by high photochemical, chemical, and thermal stabilities. It is synthesized by sintering nitrogen-rich compounds, such as melamine, urea, or thiourea, which is a simple and low-cost method. Nevertheless, GCN has a high recombination rate of photogenerated electron–hole pairs and a relatively small specific surface area, which hinders its photocatalytic performance.^14^ To improve and optimize its performance in photocatalysis, several modification techniques are used, such as metal and nonmetal doping, noble metal deposition, or synthesizing heterostructures based on GCN.^14,15^ The design of heterojunctions by coupling different semiconductors, in particular two-dimensional (2D) structures, enhances the separation of photogenerated electron–hole pairs and absorption of visible light, which promotes the photocatalytic efficiency of such materials.^16^

Layered double hydroxides (LDH) are materials based on stacked layers of octahedra, built of hydroxyl groups coordinating a central metal atom. In the octahedral layers, some of the M^2+^ cations are replaced by M^3+^ cations, which causes the layers to be positively charged. Thus, the interlayer space is occupied by hydrated anions that compensate for positive charge, usually CO_3_^2–^, NO_3_^2–^, or Cl^–^. LDH can be synthesized in several ways, including coprecipitation, hydrothermal methods, and ion exchange. During synthesis, other binary combinations of metal cations can also be prepared.^17^ Their unique properties, such as adjustable band gaps depending on the metals used during synthesis, large specific surface area, low cost, and good reusability, make them interesting as semiconductors. Additionally, their morphology and chemical and electronic properties can be easily modified by applying different synthesis methods. Still, pure LDH material’s activity in photocatalysis is often hindered by their low efficiency in utilizing light, fast recombination of electron–hole pairs, and poor dispersion in water.^18,19^ LDH/GCN heterostructures have been earlier reported as efficient materials for photocatalytic applications.^20^ Their coupling leads to the expanded light absorption range of the photocatalyst, enlarged specific surface area with an increased number of active sites, and facilitates separation of the charges.^20,21^ For instance, this includes photocatalytic CO_2_ reduction, hydrogen evolution, or degradation of Congo Red.^22−24^

In this work, the Zn–Cr LDH/GCN heterostructure was obtained with the use of three different synthesis routes. The hybrid material was designed in order to show the highest activity in visible light. In particular, this included tuning of LDH electronic properties by selection of appropriate transition metals and further heterojunction with GCN. Zinc was chosen as an M^II^ metal for LDH structure for the optimization of light absorption and high electron mobility, while Cr was selected as an M^III^ metal due to its ability to absorb a broad spectrum of visible light. The results revealed that the material obtained via the hydrothermal route contained 50% weight. GCN was characterized by the best efficiency in the photodegradation of estrone in both UV and visible light. This was due to the superior charge transfer efficiency and effectively suppressed recombination of electron–hole pairs, which induced fast degradation of the organic pollutant. In comparison to other LDH/GCN heterostructures presented in the literature,^22,25−29^ our material was prepared at a lower temperature while reaching comparable or higher current density, specific surface area, and homogeneousness. This had an important influence on materials’ photoactivity; our heterostructure was able to photodegrade pollutant in a similar or quicker time. This work gives important insight into obtaining photocatalysts through less energy-consuming paths.

## Experimental Section

### Materials

Sodium carbonate (≥99.5%, Na_2_CO_3_), sodium hydroxide (≥98%, NaOH), zinc nitrate hexahydrate (≥98%, Zn(NO_3_)_2_·6H_2_O), nitric acid(V) solution (65%, HNO_3_), diammonium oxalate monohydrate ((NH_4_)_2_C_2_O_4_·H_2_O), isopropyl alcohol (C_3_H_8_O), and ascorbic acid (C_6_H_8_O_6_) were purchased from Chempur. Melamine (≥99%, C_6_H_6_N_6_) and chromium(III) nitrate nonahydrate (≥99%, Cr(NO_3_)_3_·9H_2_O) were purchased from Acros Organics. Estrone (≥99%, C_18_H_22_O_2_) was purchased from Sigma-Aldrich. Dimethyl sulfoxide ((CH_3_)_2_SO) was purchased from Avantor. All chemicals were of analytical grade and were used without further purification.

### LDH Syntheses

Two LDH materials were obtained using different synthesis methods. In both cases, the molar ratio of Zn^II^/Cr^III^ was equal to 2:1. To obtain 1 g of each material, the general formula of LDH [M_1–*x*_^II^M_*x*_^III^ (OH)_2_][X_*x*/q_^q–^·*n*H_2_O] (M^II^ - divalent metal, M^III^ - trivalent metal, X - anion)^30^ was used to calculate the mass of substrates. The intercalated water content was not considered in the calculations. Coprecipitation method (LDH). Zinc and chromium nitrates were dissolved in 100 mL of DI water. In another beaker, a solution of 2 M NaOH and 1 M Na_2_CO_3_ was added to 80 mL of DI water to set a pH of ∼10. The solution containing metal salts was added to this beaker using a peristaltic pump (2.5 mL/min) with continuous stirring. The pH was constantly controlled at 10 (±0.1) with the use of 2 M NaOH and 1 M Na_2_CO_3_. The obtained suspension was mixed for 3 h, centrifuged, and water washed three times (4500 rpm, 3 min), then dried at 60 °C for 24 h, and ground. Hydrothermal method (HLDH). Zinc and chromium nitrates dissolved in 80 mL of water were mixed with a solution of 2 M NaOH and 1 M Na_2_CO_3_ until the pH was equilibrated at ∼10. The formed suspension was mixed for 0.5 h, and then it was placed in a glass bottle sealed with a polypropylene cap. The bottle was placed in an oven for 24 h at 100 °C. Afterward, the suspension was centrifuged and water washed three times (4500 rpm, 3 min), then dried at 60 °C for 24 h, and ground.

### GCN Preparation

Melamine was used as a nitrogen-rich substrate for the synthesis. It was placed in a closed crucible, which was followed by heating in a furnace for 5 h at 550 °C.

### Heterostructures Syntheses

These materials were obtained using 3 different synthesis routes. With the use of each method, 5 materials were synthesized: XLDH-G10, XLDH-G20, XLDH-G30, XLDH-G40, and XLDH-G50, where the number stands for the percentage of GCN in the material and X is replaced by either C, AC or H. The X symbol indicates the synthesis method: coprecipitation (C), adsorption/coprecipitation (AC), or hydrothermal (H). In all cases, the amount of substrates was calculated to obtain 1 g of the final material. Coprecipitation method. The GCN was dispersed in DI water. Then, a solution of 2 M NaOH and 1 M Na_2_CO_3_ was added to obtain a pH of ∼10. Afterward, the synthesis was conducted analogously to the LDH coprecipitation synthesis. Adsorption/coprecipitation method. The GCN was dispersed in DI water and sonicated for 5 min. Then, one drop of 1 M HNO_3_ was added to lower the pH of the suspension to ∼4. The suspension was again sonicated for 5 min. The zinc and chromium nitrates were dissolved in 100 mL of DI water and then added to the GCN suspension, sonicated for another 5 min, and stirred for 0.5 h. The low initial pH prevented the formation of instant LDH at this point. After this time, the pH was set to ∼10 with a solution of 2 M NaOH and 1 M Na_2_CO_3_. The formed suspension was mixed for 3 h, centrifuged, and water was washed three times (4500 rpm, 3 min), dried at 60 °C for 24 h, and ground. Hydrothermal method. The GCN was dispersed in DI water and sonicated for 10 min. The zinc and chromium nitrates were added to the GCN suspension followed by sonication for another 5 min and stirred for 0.5 h. Next, a solution of 2 M NaOH and 1 M Na_2_CO_3_ was added to obtain a pH of 10 (±0.1). The rest of the synthesis was conducted analogously to the LDH hydrothermal synthesis.

### Materials Characterization

The X-ray diffraction (XRD) patterns for the nonoriented powdered samples were recorded in the range of 2–75° 2θ (0.05° 2θ step) using a Rigaku MiniFlex 600 diffractometer with Cu Kα radiation. The Fourier transform infrared (FTIR) analyses were carried out with a Thermo Scientific Nicolet 6700 spectrometer in transmission mode. The spectra were collected using KBr pellets (1 wt % sample/KBr) in the 4000–400 cm^–1^ range (64 scans with 4 cm^–1^ resolution). The STEM and TEM images were obtained with an FEI Titan Cubed G2 60-300 microscope (300 kV) coupled with a high-angle annular dark-field (HAADF-STEM) and BF-STEM detector. Each sample for TEM investigations was prepared as a droplet of the water dispersion of each material (sample) that was placed on the carbon grid and vacuum-dried. The examination of the samples’ specific surface area was carried out based on low-temperature N_2_ adsorption/desorption measurements (77 K) using the ANTON PAAR NOVA 800 instrument. Prior to analysis, the samples were outgassed at 105 °C for 24 h. The specific surface area (*S*_BET_) was calculated according to the BET equation. The X-ray fluorescence spectroscopy (XRF) was performed with the use of the Rigaku ZSX Primus II apparatus equipped with the Rh lamp. The CHN elemental analysis was carried out through the combustion of samples and the measurement of purified and separated gaseous products using a VarioEL III Elementar analyzer. The X-ray photoelectron spectroscopy (XPS) spectra were recorded by a Thermo Fisher spectrometer using monochromatic Al Kα radiation (1486.6 eV). The peak fitting was performed by using Fityk software. The optical properties of the samples were determined with the use of the spectral dependence of the total reflectance obtained with a Jasco V670 double-beam UV–Vis/NIR spectrophotometer. The Kubelka–Munk function was used to determine the energies of band gaps for the heterostructures.^31^ The photoluminescence (PL) and time-resolved photoluminescence lifetime (TRPL) measurements were obtained on the FL920 Edinburgh Instruments spectrophotometer with excitation and emission wavelengths of 350 and 467 nm, respectively.

### Photoelectrochemical Measurements

The CHI 7273D electrochemical workstation with a standard three-electrode photoelectrochemical cell was used for the measurements. An Ag/AgCl electrode and platinum plate were used as a reference and counter electrode, respectively. The catalyst ink was prepared by dispersing 5 mg of analyzed material in a mixture of 600 μL of DI water, 200 μL of ethanol, and 20 μL of Nafion (5 wt %), followed by 20 min of sonication. The ink was deposited on a Fluorine Doped Tin Oxide (FTO) plate using a drop-casting method on a 1 × 1 cm^2^ area and used as a working electrode. For the linear sweep voltammetry (LSV) and electrochemical impedance spectroscopy (EIS), 0.5 M Na_2_SO_4_ solution was used as an electrolyte. In photocurrent measurements, 1 M KOH electrolyte was used, and the working electrode was examined with chopped light voltammetry for several on–off cycles of light irradiation with the use of a 300 W Xe lamp (Perfect light PLS-SXE300) with a UV-cutoff filter.

### Photocatalytic Experiments

As a source of visible light, a 150 W LED lamp with a light illuminance of approximately 120 000 lx was used. Photodegradation in UV light was performed by using a 35 W UV lamp (365 nm peak emission wavelength) with a polypropylene filter to adjust the light power density to around 10 mW/cm^2^. The temperature inside the reactor at the end of the experiments was below 35 °C. The materials’ photocatalytic activity was tested in batch experiments in aqueous solutions containing 1 ppm of estrone at room temperature. The suspension density (i.e., catalyst dosage) was equal to 0.5 g/L (50 mg per 100 mL). After 5 min of mixing the suspension, the light was turned on and the experiment was conducted for 1 h. The supernatants were collected through centrifugation (18 000 rpm, 3 min) every 10 min. Control experiments were performed in the dark, to assess the adsorption rate and efficiency. The pH of the estrone solution was 6.3, and after the addition of the obtained materials, the pH remained basic, ranging between 8 and 10. The estrone concentration was measured with the use of HPLC with Shimadzu LC-2050C with a UV detector. The conditions of the measurements are provided in the Supporting Information. Additionally, the reuse and stability of LDH, HDLH, GCN, CLDH-G50, ACLDH-G50, and HLDH-G50 materials were further examined in visible light. Their selection was based on the performance evaluated in preceding experiments. A total of 4 cycles of photocatalytic reactions were performed for each material. The supernatants were collected after 60 min of photocatalysis. After every experiment, the photocatalyst was washed with 50 mL of distilled water and dried. The materials’ chemical stability was calculated based on Zn and Cr concentrations in supernatants, measured with AAS using the GBC SavantAA instrument. For the 6 materials mentioned above, active species possibly involved in photodegradation were detected using different scavengers. Dimethyl sulfoxide (DMSO), isopropyl alcohol (IPA), ammonium oxalate (AO), and ascorbic acid (AA) were used as trapping agents for electrons, hydroxyl radicals, holes, and superoxides, respectively.^32,33^ The reaction conditions were the same as those previously mentioned, with the final 5 mM concentration of trapping agents in the pollutant solution. For experiments with AA, a lower concentration of 0.1 mM was used to maintain the pH above 4.

## Results and Discussion

### Materials Characterization

**Figure 1:**
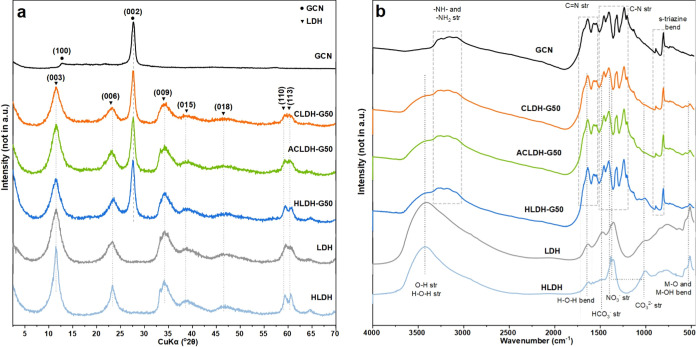
(a) XRD patterns and (b) FTIR spectra of the GCN, LDH, HLDH, CLDH-G50, ACLDH-G50, and HLDH-G50 samples.

The XRD patterns of heterostructures obtained by different synthesis routes were compared in Figure 1a, while a comparison between materials with different GCN content was presented in Figure S1. The GCN material has two diffraction peaks at 12.83 and 27.65°, which correspond to (100) and (002) lattice planes, respectively.^34^ The diffraction peaks for LDH and HLDH visible at 11.50, 23.26, 34.23, 39.08, 46.61, 59.39, and 60.62° were ascribed, respectively, to (003), (006), (009), (015), (018), (110) and (113) planes.^35^ The LDH reflections in the XRD pattern of the obtained heterostructures remained at the same positions. As for the GCN reflections, the first at 12.83° was hardly visible due to overlapping with the LDH peak at 11.50°. However, the main GCN reflection (27.65°) was visible, and its intensity varied with the GCN content reflecting the LDH to GCN ratio. With the increase of GCN content in the materials, the intensity of LDH reflections decreased, opposite to the intensity of the GCN main reflection. Moreover, the main GCN peak was slightly shifted, in particular in the case of hydrothermal heterostructures, and its relative intensity as compared to 11.50° of LDH varied depending on the material (Figure S1). This might result from the mutual impact of individual phases on the stacking behavior of particles in the composite material as noticed previously.^22^ The analysis of the FWHM values calculated for the main LDH reflection (11.50°) showed differences between materials (Table S1). The materials obtained by adsorption/coprecipitation had slightly sharper reflections (average FWHM: 2.14 ± 0.2) than the materials obtained solely by precipitation (average FWHM: 2.29 ± 0.09). While both the heterostructures (average FWHM: 1.95 ± 0.35) and the pure LDH obtained by hydrothermal synthesis (FWHM: 1.24) were characterized by even sharper reflections. This suggests they formed larger crystallites and/or indicated their higher structural order. The peaks were gradually sharpening with the decrease of GCN content, which indicated that the presence of GCN during the synthesis affected the LDH crystallinity and crystal size. Slightly higher crystallinity of hydrothermally obtained materials can lead to enhanced photoactivity, due to faster transportation of charge carriers, more efficient light absorption, and improved structural integrity, while still possessing a high number of defects and grain boundaries for trapping the carriers, which elongates recombination rate.^36,37^

In terms of textural parameters, GCN was characterized by the lowest *S*_BET_ of 13.0 m^2^/g. The *S*_BET_ of the HLDH (192.2 m^2^/g) was over 2 times higher than that of the LDH (91.6 m^2^/g). As for the heterostructures, the average *S*_BET_ values were lowest for the materials obtained by coprecipitation and were equal to 85.6 ± 13.0 m^2^/g. The average specific surface for materials obtained by the adsorption/coprecipitation method was slightly higher (95.1 ± 12.7 m^2^/g), while the average *S*_BET_ for the hydrothermally synthesized materials was the highest (133.1 ± 20.2 m^2^/g). This trend along with FWHM calculations clearly indicated that the materials obtained hydrothermally have better developed porosity than the materials obtained by coprecipitation or adsorption/coprecipitation. It is worth noting that the main contribution comes from the HLDH phase.

A comparison of the FTIR spectra of heterostructures obtained by different synthesis routes, as well as pure materials, is presented in Figure 1b. FTIR spectra comparison for the heterostructures with different GCN content was displayed in Figure S2. In the GCN spectrum, several bands were observed around 3000–3300 cm^–1^, coming from the N–H stretching vibrations. The bands around 1520–1650 and 1200–1480 cm^–1^ were assigned to C=N and C–N stretching vibrations of the aromatic repeating units, respectively. The bands at 807 and 889 cm^–1^ were ascribed to the characteristic out-of-plane vibrations of s-triazine aromatic repeating units.^38,39^ The wide band observed at 3420 cm^–1^ for the LDH was associated with the O–H stretching vibrations in the octahedra and also the H–O–H stretching vibrations of the bound water. The band at 1636 cm^–1^ was ascribed to the H–O–H bending vibrations of bound water. The bands at 1480, 1383, and 1355 cm^–1^ were assigned to stretching vibrations of the interlayer anions: HCO_3_^–^, NO_3_^–^, and CO_3_^2^, respectively.^40−42^ The bands around 500 cm^–1^ were coming from the lattice vibration modes of the M–O and M–OH vibrations.^42,43^ The spectrum of HLDH showed the same bands as for LDH, and an additional relatively broad band at 1003 cm^–1^ coming from CO_3_^2–^. This band is activated due to lowering the anion symmetry (site group splitting).^42,44^ The band appeared due to the higher concentration of CO_3_^2–^ anions in the interlayer space. This is linked to a lower concentration of HCO_3_^–^ anions, which is evidenced by the lower intensity of its band compared to the LDH material. Carbonates in the interlayer space might improve the photocatalytic activity of materials, due to the generation of charge carriers, reducing recombination rate, and facilitation of the reaction between reactant molecules.^45^ The spectra of heterostructures contain bands from both LDH/HLDH and GCN. As the content of GCN material increased, its bands became more intense. The bands’ profiles including relative intensities and positions did not differ from the spectra of pure components, confirming the formation of composites with features similar to a physical mixture.

**Figure 2:**
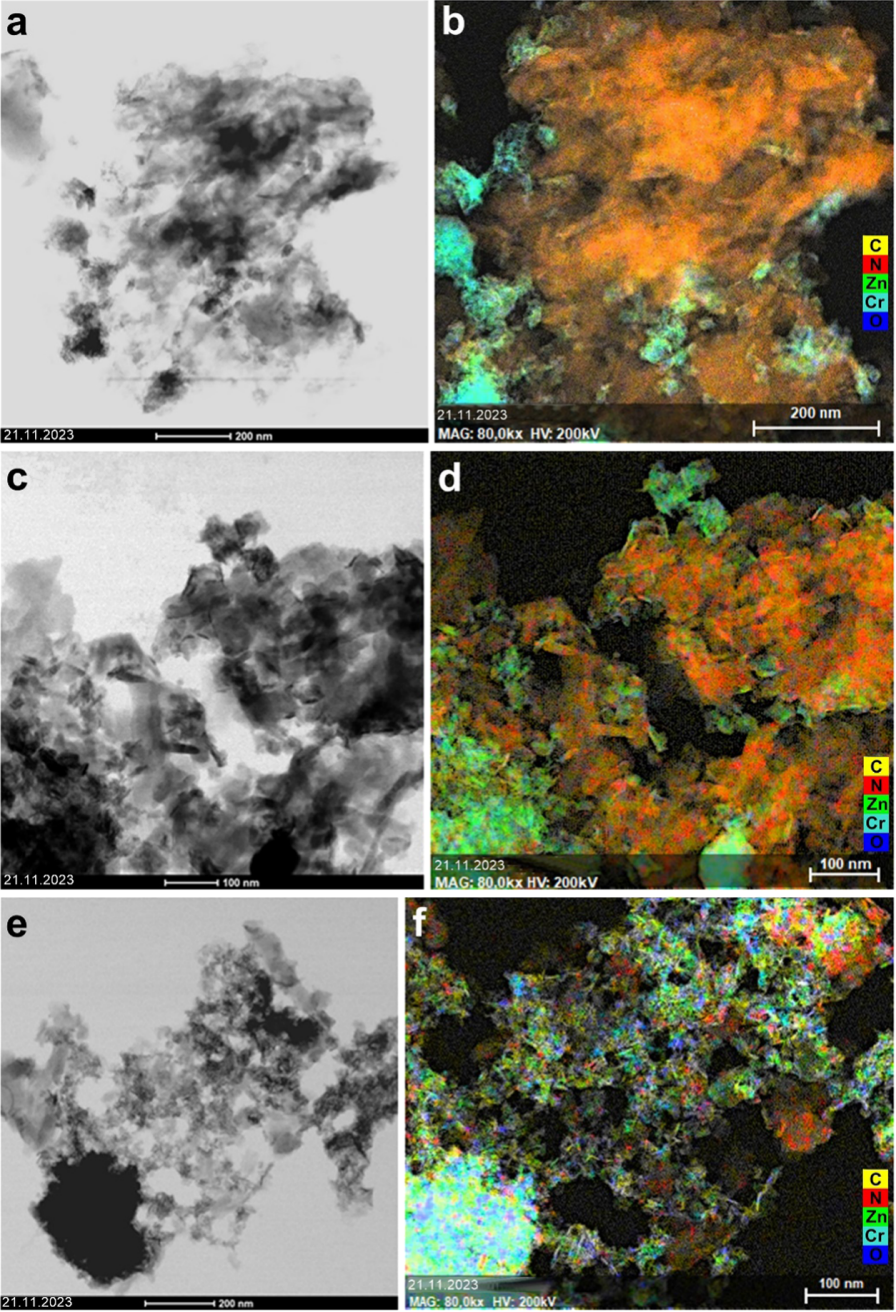
BF-STEM images and EDS maps of the: (a, b) CLDH-G50, (c, d) ACLDH-G50, and (e, f) HLDH-G50.

The TEM images of LDH, HLDH, GCN, CLDH-G50, ACLDH-G50, and HLDH-G50 are presented in Figure S3. In the samples containing LDH obtained by the coprecipitation (Figure S3a,d,e), aggregated plate-like nanoparticles without distinct features are observed. In the case of hydrothermal materials (Figure S3c,f), aggregated thin flakes forming house of cards structures characteristic for the LDH are visible. The TEM image of GCN (Figure S3b) shows aggregates of 2D elongated sheets with irregular morphology. As can be seen from EDS mapping, for the CLDH-G50 material (Figure 2a,b) the distribution of LDH particles is uneven, with LDH clusters located mostly on the edges of the GCN aggregates. For the ACLDH-G50 (Figure 2c,d) it is observed that the LDH particles are more evenly distributed, and the LDH flakes are better defined. In turn, for the HLDH-G50 (Figure 2e,f) the GCN material is coated with the well-crystallized LDH flakes, forming the most homogeneous heterostructure. The TEM images, along with EDS mapping, support the conclusions from the XRD and *S*_BET_ analyses. The crystallinity of the LDH in heterostructures is increasing in the following order: CLDH-G50, ACLDH-G50, HLDH-G50. Due to the morphology of the LDH phase in the HLDH-G50 heterostructure, this material is characterized by the most porous structure, which results in the highest specific surface area.

The XPS analysis was performed to get insight into the surface chemical composition of the pure materials (GCN, LDH, and HLDH) and 3 composites (CLDH-G50, ACLDH-G50, and HLDH-G50). The N 1s spectrum for pure GCN was deconvoluted to 3 peaks (Figure S4a). They are located at binding energies of 398.3, 399.6, and 400.7 eV and ascribed to sp^2^ hybridized nitrogen (C=N–C), which confirmed the formation of s-triazine structure, bridged tertiary nitrogen groups (N–C_3_), and surface amino functional groups (C-NH_*x*_), respectively.^22,46,47^ The C 1s spectrum of pure GCN was fitted with 3 peaks at: 284.3, 287.8, and 288.9 eV (Figure S4b). They correspond to C=C bonds of the trigonal C–N network, sp2 hybridized carbons in the s-triazine ring (N–C=N), and C–O bonds on the surface of the material, respectively.^47,48^ The presence of C–O bonds was previously reported^49^ and corresponds survey XPS spectrum of pure GCN. As for N 1s and C 1s spectra for the heterostructures, all peaks fitted for the pure GCN remained and were shifted toward higher binding energies. This observation indicates a decrease in the electron densities of nitrogen and carbon atoms. This can be related to the strong interaction of the heterostructure components and subsequent interfacial charge transfer effects.^23,50^ The Zn 2p spectra for all analyzed materials show 2 peaks: Zn 2p_3/2_ and Zn 2p_1/2_ (Figure S4c). Both peaks were subsequently deconvoluted into 2 peaks corresponding to Zn–O and Zn–OH bonds.^50−52^ The Zn 2p_3/2_ peak for LDH was fitted to 1022.3 and 1024.6 eV peaks (Zn–O and Zn–OH, respectively), while the Zn 2p_1/2_ peak was deconvoluted into 1045.3 and 1047.3 eV peaks (Zn–O and Zn–OH, respectively). For all materials, the spectra of Cr 2p had 2 broad peaks visible ascribed to Cr 2p_3/2_ and Cr 2p_1/2_ (Figure S4d). Again these peaks were fitted with 2 peaks, corresponding to Cr–O and Cr–OH bonds.^53−55^ For the LDH, the Cr 2p_3/2_ peak was deconvoluted to 577.8 and 580.4 eV peaks (Cr–O and Cr–OH, respectively), and the Cr 2p_1/2_ peak was deconvoluted to 587.4 and 590.8 eV peaks (Cr–O and Cr–OH, respectively). For the LDH material, the O 1s spectrum was deconvoluted to 4 peaks (Figure S4e). The peak at 530.9 eV corresponds to lattice oxygen (M–O), while the strongest peak at 523.0 eV is attributed to oxygen bridges (M–O–M). The 533.6 eV peak is linked to surface hydroxyl groups (M–O–H) and the peak at 535.9 eV comes from the presence of water in the LDH interlayer (H–O–H).^56−58^ The HLDH material exhibited peak shifts to lower binding energies in all spectra, in comparison to the coprecipitated LDH. This indicates a higher density of electrons in the HLDH material and suggests that LDH materials obtained by coprecipitation exhibit a different charge distribution in their structure than the LDH obtained by the hydrothermal method. For both CLDH-G50 and ACLDH-G50 heterostructures, a shift in all peaks to the lower binding energies is observed, in comparison to the LDH. This states the higher electron densities for Zn, Cr, and O, which, along with the lower electron densities observed for N and C, confirms the interfacial charge transfer effects within the formed heterostructures.^22,56^ The XPS spectrum analysis for the HLDH-G50 heterostructure is more complex. The Zn 2p peaks exhibited a shift to higher binding energies in comparison to the HLDH. In turn, for the Cr 2p peaks, the Cr–O peaks were shifted to higher binding energies and the Cr–OH peaks were shifted to lower binding energies. The O 1s spectrum was characterized by M–O and M–O–M peaks shifting to higher binding energies and M–O–H and H–O–H peaks shifting to lower binding energies. These observations, along with peaks shifting to higher binding energy values for C 1s and N 1s spectra, suggest the complex electronic structure of the HLDH-G50 material compared to the other composites. This may result in better separation of the induced charge carriers, which promotes the photocatalytic activity of the material as shown in the following experiments.

The results of the XRF and CHN measurements for the obtained materials are presented in Table S3. The Zn/Cr ratios calculated for LDH and HLDH were equal to 2.23 and 2.43, respectively. These values were higher than the assumed Zn/Cr ratio of 2.0 and indicated a lower structural charge due to the loss of chromium in particular for the HLDH material. The heterostructures showed slightly lower Zn/Cr ratios as compared with the adequate LDH material but still higher than the theoretical value. The average Zn/Cr ratio values for the materials obtained by coprecipitation and adsorption/coprecipitation were equal to 2.11 ± 0.04 and 2.12 ± 0.12, respectively. For the hydrothermal heterostructures, the mean Zn:Cr ratio was 2.25 ± 0.04. These results suggest that Cr ions are slightly more abundant in the LDH structures obtained in the presence of GCN. Since Cr^3+^ ions provide better photoresponsiveness in visible light,^59^ this may contribute to better photoactivity of the heterostructures, compared to the pristine LDH and HLDH. The theoretical content of C and N in GCN should be equal to 39.1 and 60.9 wt %, respectively (N/C ratio = 1.55). The CHN measurements for this material showed slightly different results for C and N content (N/C ratio = 1.79), due to the incomplete condensation of s-triazine units.^60,61^ This was caused by the preparation of the material in the presence of oxygen. The presence of C and N in the LDH and HLDH materials is caused by carbonates and nitrates present in the interlayers of the structures. These values are consistent with FTIR spectra of LDH and HLDH, indicating that carbonates are dominant interlayer anions. For all heterostructures, the N/C ratio was lower than that for the pristine materials. A relationship can be observed in which the N content decreased more significantly with an increase of the LDH content in the heterostructure. The reason for that is that the lower the content of the GCN material in the heterostructure, the greater the influence of carbonates on the total C content. The GCN content was calculated by subtracting the calculated nitrate content from the total N content for each heterostructure and comparing it to the N content in the pure GCN. All of the obtained heterostructures have slightly lower GCN contents than the assumed values. However, it is less than 5 pp. for all cases and probably is caused by removing some of the smallest GCN particles during washing the materials after syntheses.

### Band Gap Analysis

**Figure 3:**
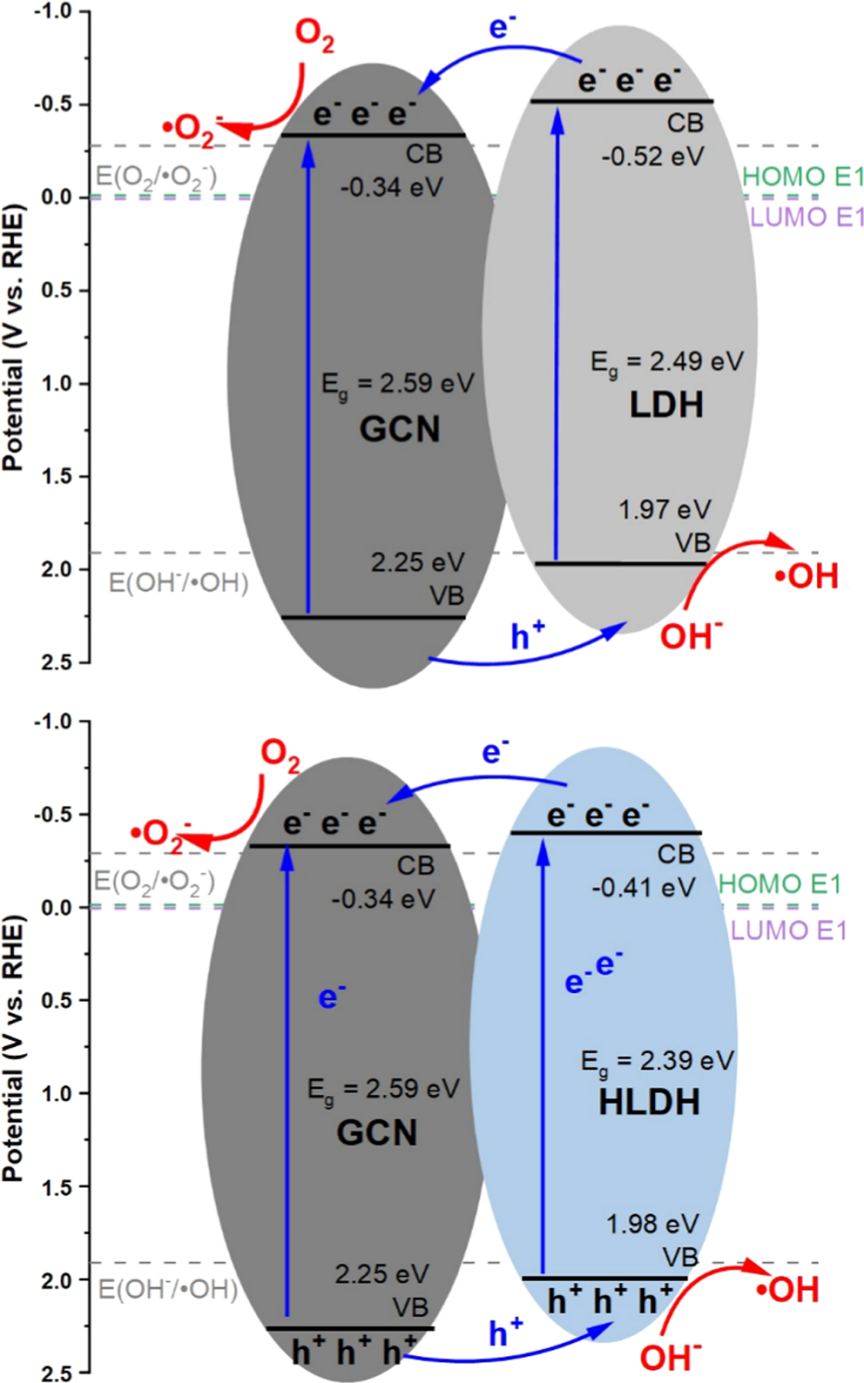
Proposed mechanism driving photodegradation of estrone by the GCN/LDH and GCN/HLDH heterojunctions.

The optical band gaps (Figure S5a–c) were calculated with the use of Tauc’s equation, from the linear part at the lower photon energy region.^31^ To determine the valence band (VB) and conduction band (CB) energies, the right slopes of the VB-XPS spectra (Figure S5d–f) were extrapolated to the baseline. The GCN band gap was indirect and determined to be 2.59 eV. The VB and CB edge positions were estimated to be 2.25 and −0.34 eV, respectively. The results are consistent with the structure of GCN in which triazine is the linker in the structure.^62^ As for the LDH and HLDH, they were characterized with multifaceted band structures due to the existence of different types of electronic transitions in the materials. For the LDH and HLDH, 3 different direct band gap energies were determined: 1.50 eV (E_g1_), 2.49 eV (E_g2_), 3.97 eV (E_g3_), and 1.61 eV (E_g1_), 2.39 eV (E_g2_), 4.00 eV (E_g3_), respectively. The E_g1_ and E_g2_ are linked to the d-d transitions of the Cr^3+^ ion, while the E_g3_ is linked to the O–Zn and O–Cr electron transitions.^63,64^ The E_g2_ is the energy considered further since the carriers from the narrowest band gap energies experience fast recombination of charges.^59,63^ The VB and CB edge positions were calculated to be 1.97 and −0.52 eV for the LDH and 1.98 and −0.41 eV for the HLDH. The anticipated mechanisms of estrone photodegradation with the LDH/GCN heterostructures were presented in Figure 3. The VB maximum and CB minimum of LDH and HLDH are higher than those for the GCN suggesting the formation of type II heterojunctions.^65^ Both CBs have lower negative potential than that of O_2_/^•^O_2_^–^ (0.33 V) and VBs display greater positive potential than that of OH^–^/^•^OH (1.91 V). Due to that, the charge transfer mechanisms were complex and it was not possible to determine if the Z-type heterojunction occurs with the use of the scavengers experiments.^66^ Thus, the proposed mechanism for the obtained materials was staggered heterojunction. The visible light irradiation causes the excitation of electrons and the generation of holes both in the GCN and LDH/HLDH, due to their band gap energies, which lie in the visible light region. The excited electrons in the CB of LDH/HLDH can also pass through the heterojunction into the CB of GCN, while the formed holes of the GCN VB can pass across the heterojunction to the VB of LDH/HLDH. These processes promote charge separation and enhance the photocatalytic efficiency of the heterostructures. The holes in the VB of GCN and LDH/HLDH can induce the formation of hydroxyl radicals, while electrons in the CB of GCN and LDH/HLDH can produce superoxide radicals, which take part in the degradation of estrone molecules. The observed lower band gap energy of HLDH in comparison to that of LDH may enhance the photodegradation rate. The difference in band gap energies between LDH and HLDH occurs due to the structural, morphological, and electronic differences between these materials. As it was stated based on XRD and FTIR results, there are differences in crystallinity and homogeneity of the LDH/HLDH phases, and different ratios of nitrates to carbonates in their interlayer environment. The HLDH appeared also to have a more favorable arrangement of metal atoms in the structure, according to XPS, which all led to lowering of its band gap. Additionally, in Figure 3 HOMO (−0.013 eV) and LUMO (0.006 eV) the energy states of estrone were presented.^67^ Electrons can be excited from the E1 HOMO to its LUMO under light irradiation. Due to the CB edge of the GCN lies beneath the LUMO of E1, the electrons might be transferred from E1 LUMO to CB of the GCN. This transfer is thermodynamically favorable because the electrons will move from a higher energy state (0.006 eV) to a lower one (−0.34 eV). Similarly, holes generated in the LDH/HDLH valence band (1.97/1.98 eV) can be transferred to the estrone’s HOMO (−0.013 eV). However, the large energy difference (1.99 eV) suggests that this process might be less efficient unless intermediate states or additional mechanisms facilitate it.

### Photoelectrochemical and Photoluminescence Properties

**Figure 4:**
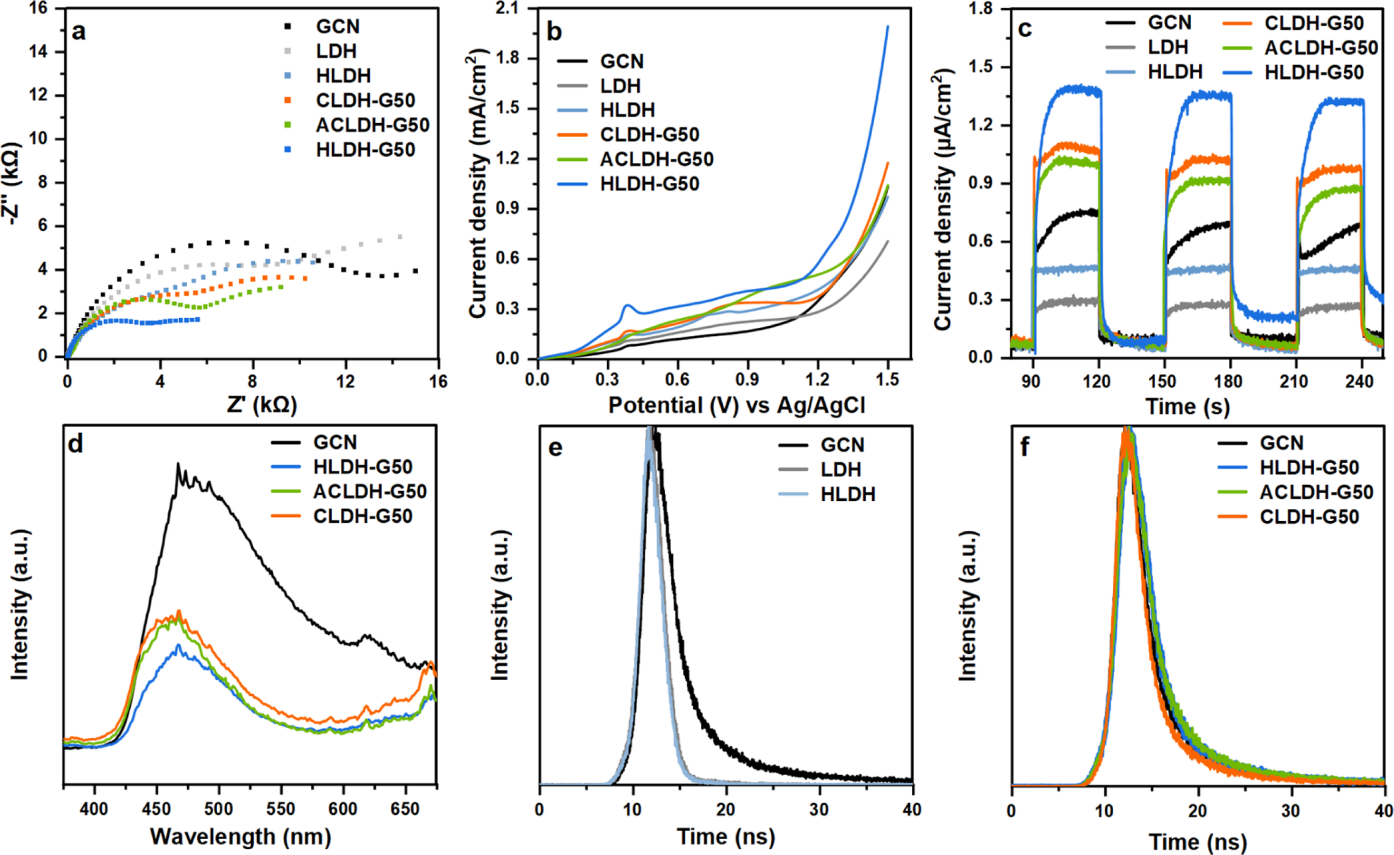
(a) Nyquist plots, (b) LSV curves, (c) photocurrent measurements in on–off cycles, (d) PL spectra, and (e, f) TRPL spectra of the GCN, LDH, HLDH, CLDH-G50, ACLDH-G50, and HLDH-G50.

For further understanding of the photocatalytic mechanisms for the heterostructures, photochemical measurements were performed. The Nyquist plots obtained from the EIS measurements for the heterostructures and the pristine materials are presented in Figure 4a. The charge transfer resistance (*R*_ct_) across the electrode/electrolyte system is expressed with the diameter of the arc radius in the semicircle recorded for the electrode material.^68,69^ As can be seen, the charge transfer resistance decreases in the following order: GCN, LDH, HLDH, CLDH-G50, ACLDH-G50, and HLDH-G50. These results revealed that the introduction of LDH and its coupling with the GCN structure led to a reduction of charge transfer resistance and shortened ion diffusion pathway distance and time. The LSV curves indicated photocurrent density values (at 1.5 V vs Ag/AgCl) which increased in the following sequence: LDH (0.71 mA/cm^2^), HLDH (0.98 mA/cm^2^), GCN (1.04 mA/cm^2^), ACLDH-G50 (1.05 mA/cm^2^), CLDH-G50 (1.18 mA/cm^2^), and HLDH-G50 (2.00 mA/cm^2^) (Figure 4b). The same order of generated photocurrent density was observed for the materials in the on–off cycles (Figure 4c). All of the materials exhibited a rapid increase in photocurrent upon illumination, which indicated efficient charge carrier generation and transport. When the photocatalyst is illuminated, current flows through directly, which results in the shape of the photocurrent response, as seen for the LDH and HLDH materials. However, the characteristic gradual current increase shape observed in the initial phase of recorded response for the GCN and heterostructures suggests the existence of holes and traps in their structures.^53^ These are responsible for prolonging the carriers’ lifetimes and thus suppressing electron–hole recombination, which leads to induced electron current.^70^ The heterostructures exhibited slower loss of current density after the light was turned off, which can be seen in time frames 120–150 and 180–210 s. For these materials, the shapes of chopping graphs were arc-type, in contrast to the pristine materials, which exhibited instant loss of current after turning off the light. This is also an indication of the presence of holes and traps in the heterostructures and a fast recombination rate in the pristine materials. The results of the photochemical measurements suggested superior electronic conductivity and charge transfer efficiency for the hydrothermal heterostructure. It exhibited the highest photocurrent density in steady state of all analyzed materials, slowest loss of photocurrent in the dark, and the most curved shape of an arc under light illumination. The obtained results correlate well with the structural and electronic properties of the materials discussed above. These factors are crucial for the photocatalytic efficiency of materials due to the higher mobility of charge carriers and their more rapid transport to the surface of the material, which reduces the recombination of electron–hole pairs and thus improves degradation of the pollutant. High efficiency of charge transfer also enables the generation of charge carriers by lower-energy photons, broadening the usable portion of the visible light spectrum.^71,72^

It is commonly known that the response observed in the PL spectra depends on the recombination of photoinduced electron–hole pairs, which can reveal materials’ ability to separate and transform photogenerated charge carriers (Figure 4d). The GCN spectrum was characterized by the highest intensity, and all 3 spectra of heterostructures had significantly lower intensities. The smallest intensity and hence the lowest recombination rate was recorded for the HLDH-G50. These results agreed well with the photocatalytic activity of heterostructures and electrochemical studies.

The TRPL spectra shown in Figure 4e,f were collected to analyze the dynamics of charge carrier transfer in time. The decay curves for the GCN and heterostructures were fitted with the use of biexponential kinetic function (eq 1). As for the decay curves of LDH and HLDH, an exponential kinetic function was used in fitting (eq 2). The average lifetime was also calculated according to eq 3.^32^
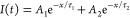
1
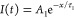
2
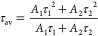
3The relative amplitudes of the decay species (*A*_1_ and *A*_2_), the charge carrier lifetimes (τ_1_ and τ_2_), and the average lifetime (τ_av_) are presented in Table S4. The average carrier lifetimes of HLDH-G50 and ACLDH-G50 were 2.96 and 3.00 ns, respectively. The values were almost 2 times higher than the lifetimes of pure LDH (τ_av_ = 1.52 ns) and HLDH (τ_av_ = 1.54 ns). The calculated average lifetimes for both of these heterostructures’ were also longer than the one calculated for the GCN equal to 2.64 ns. The CLDH-G50 heterostructure was characterized by an average lifetime equal to 2.52 ns, which was longer than that for pure LDH, but shorter than that for GCN. The short lifetime (τ_1_) is attributed to the radiation process of electron–hole recombination. The long lifetime (τ_2_) reflects the nonradiation energy transfer processes coming from the indirect formation of self-trapped excitons, due to the presence of defects in the semiconductor structure.^23,73^ The biexponential function did not give a good fit with the decay curves of the pure LDH and HLDH. This suggested that these materials contain negligible amounts of traps that can prevent charge recombination. The τ_1_ values of both HLDH-G50 (2.94 ns) and ACLDH-G50 (2.98 ns) were found to be longer than that of GCN (2.54 ns). The τ_2_ of all 3 heterostructures was calculated to be longer than that of the GCN (16.80 ns): 20.15, 22.00, and 23.47 ns for the CLDH-G50, ACLDH-G50, and HLDH-G50, respectively. These results agreed well with electrochemical studies and indicated that the formed heterostructures effectively suppressed the recombination rate of the photoinduced charge carriers. Due to this, a high probability exists of their participation in a series of photocatalytic reactions on account of the traps in their structures.^74^ These reactions fasten the photodegradation rate of the pollutant, which is essential for an effective photocatalyst.

### Photocatalytic Experiments

**Figure 5:**
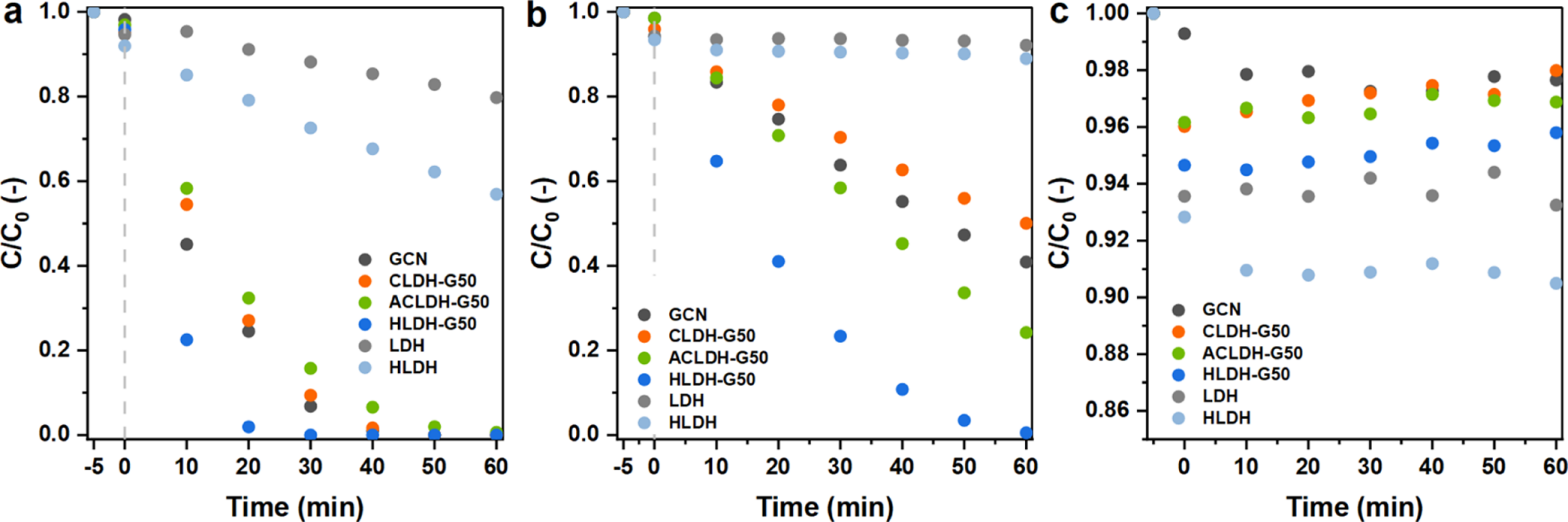
GCN, LDH, HLDH, CLDH-G50, ACLDH-G50, and HLDH-G50 kinetics of (a) UV light photodegradation, (b) visible light photodegradation, and (c) adsorption of estrone performed in the dark.

Prior to the photodegradation study, the photolysis of estrone in UV and visible light was tested. These studies showed that less than 5% of the estrone concentration degraded in UV and less than 0.5% degraded in visible light. The photocatalytic activities in UV and visible light of all synthesized samples were presented in Figure S6, along with adsorption rates as a control experiment performed in the dark. All of the tested materials exhibited a relatively small estrone adsorption capacity. After 60 min, the adsorption rate was less than 3% for the GCN, less than 7% for the LDH, and less than 10% for the HLDH. With regard to all of the heterostructures, not more than 5% of estrone was adsorbed after 60 min of the reaction. The materials’ active specific surface area described above did not have a strong effect on the adsorption capability, due to the nonpolarity and hydrophobicity of estrone molecules. As shown in Figure 5c and Table S5, the difference in adsorption rate between materials with the highest and lowest *S*_BET_ value (HLDH and GCN, respectively) was less than 8 pp.

In both UV and visible light, the heterostructures with a higher GCN content generally exhibited higher photocatalytic activity (Figure S6). A comparison of photocatalytic activity in UV and visible light between the pure materials and 3 types of heterostructures (containing 50% of GCN) is presented in Figure 5a,b. In both experiments, the HLDH-G50 material exhibited the highest photoactivity. In UV light, after 30 min, the estrone concentration was below the detection limit. In comparison, a 93.2% concentration loss was measured for the GCN. The other 2 heterostructures, CLHD-G50 and ACLDH-G50 were characterized by 90.6 and 84.2% concentration loss, respectively. The LDH and HLDH materials exhibited only 11.8 and 27.4% concentration loss, respectively. In the experiments with visible light, the photocatalytic activity of pure LDH and HLDH after 60 min was negligible considering the adsorption rate and equal to 7.9 and 11.0%, respectively. Photodegradation of estrone with the use of CLDH-G50, ACLDH-G50, and GCN resulted in 49.9, 75.8, and 59.1% concentration loss in 60 min, respectively. While the HLDH-G50 showed much greater efficiency, which led to a concentration loss of 99.5%. These results indicate that the formed heterostructures can be used for the photodegradation of organic pollutants in both UV and visible light. This is attributed to their light absorption properties and the appropriate band gap energies described above. The hydrothermal heterostructure exhibited superior photocatalytic efficiency, which is consistent with the analysis of its electronic structure and morphology. It is worth underlining the synergistic effect of GCN and the hydrothermal LDH heterojunction, which is especially pronounced in the visible light region. The efficiency and removal rate of estrone are strongly influenced by the applied experimental conditions. The photodegradation efficiency depends on factors such as catalyst dosage, pH of the solution, and the wavelength and power of used light. Additionally, different concentrations were used in the reported papers, which makes a comparison of results difficult. For example, in the work presented by Zhu et al.,^75^ 94.9% of estrone (initial concentration *C*_in_: 2.8 ppm) was removed in 1 h under UV–vis light irradiation. In a paper presented by Zhang et al.,^76^ 45% of estrone (*C*_in_: 0.001 ppm) was degraded after 1 h exposure in a UV reactor. In turn, a photocatalyst described by Sornalingam et al.^77^ was able to photodegrade ∼95% of estrone (*C*_in_: 1 ppm) in visible light (“cool white” LED). In another research reported by Farooq et al.,^78^ estrone was not fully degraded even after 3 h of visible light irradiation (*C*_in_: 1 ppm). In our work, after 1 h of visible light irradiation, 99.5% of estrone with a *C*_in_ of 1 ppm was degraded. This suggests the high efficiency of our material in relation to other photocatalysts presented in the literature.

**Figure 6:**
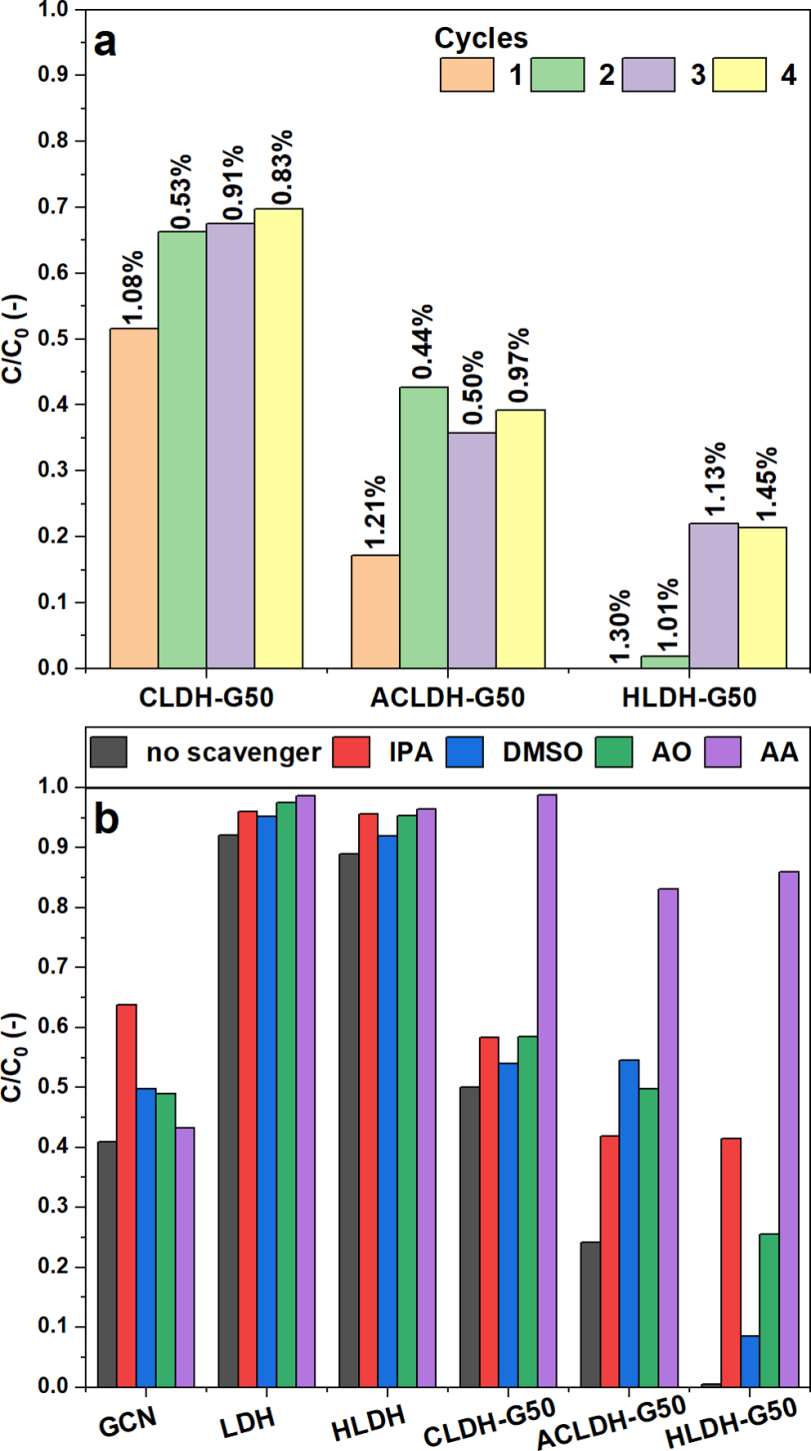
Results showing: (a) reusability and stability studies of the heterostructures and (b) estrone degradation efficiency in the presence of different scavengers. The values above bars indicate the percentage of LDH material that was dissolved during experiments.

The experiments on heterostructures’ recyclability are presented in Figure 6a. After 4 cycles, the CLDH-G50 material exhibited 30.2% of E1 concentration loss, which was 18.2 pp. less than in the first cycle. In turn for the ACLDH-G50, in the fourth cycle, the concentration loss was equal to 60.8%, which was 21.9 p.p. less than in the first cycle. The HLDH-G50 material was characterized by 78.7% of E1 concentration loss in the fourth cycle which is 21.3 p.p. less than in the first cycle. This shows that the materials can be successfully reused for the efficient removal of estrone. The changes in efficiencies for each material between the cycles emphasize the importance of thoroughly rinsing the material after each cycle. This also suggested that different regeneration methods may be implemented to improve heterostructures’ reusability. As shown in Figure 6a, the amount of LDH that was dissolved during each photocatalytic experiment did not exceed 1.5%. This suggested high stability of heterostructures under the applied experimental conditions, which, however, can be further improved by changing the synthesis conditions.

The photocatalytic mechanisms behind estrone degradation were further investigated with the use of different scavengers. Figure 6b shows the involvement of the active species in the estrone photodegradation process. The isopropyl alcohol (IPA), dimethyl sulfoxide (DMSO), ammonium oxalate (AO), and ascorbic acid (AA) were used as scavengers of hydroxyl radicals (^•^OH), electrons (e^–^), holes (h^+^), and superoxide radicals (^•^O_2_^–^), respectively. The experiments showed that the hydroxyl radicals were the key reactive species in the case of GCN, while the other species played less important roles in the photodegradation of estrone. For both LDH and HLDH, all of the active species were similarly involved in the photocatalytic process. As for the heterostructures, the dominant reactive species were superoxide radicals. Their suppression by ascorbic acid significantly lowered the degradation efficiency. All of the other reactive species had a lower impact on the photodegradation process; however, their contribution visibly differed for the heterostructure. The influence of active species can be shown in the following order: ^•^O_2_^–^ > h^+^ ≥ ^•^OH > e^–^ for the CLDH-G50, ^•^O_2_^–^ > e^–^ > h^+^ > ^•^OH for the ACLDH-G50 and ^•^O_2_^–^ > ^•^OH > h^+^ > e^–^ for the HLDH-G50. This experiment proved that the synthesis conditions of the heterostructures strongly affect the mechanisms of estrone photodegradation. Additionally, since superoxide radicals were dominant active species, it suggested that the use of additional electron acceptors (such as O_2_ or H_2_O_2_) in the system may strongly improve the photocatalytic performance of heterostructures.^76^

## Conclusions

In summary, 3 variations of LDH/GCN type II heterostructures were successfully formed with the use of different synthesis methods. All of them exhibited complex charge transfer mechanisms and reduced recombination rates in comparison to the pristine GCN and LDH/HLDH materials. Their structure, morphology, and electronic properties differed, which influenced their photocatalytic activity toward estrone degradation. The HLDH-G50 material, obtained via a hydrothermal route and containing 50% of GCN had the best efficiency in photodegrading estrone. In visible light, 99.5% of estrone was degraded after 1 h, while in UV light, the estrone concentration was below the detection limit after 30 min. This material was characterized by high crystallinity and porosity, which contributed to its high specific surface area. The carried out electrochemical and photoluminescence studies revealed that the HLDH-G50 material had a complex electronic structure, generated the highest photocurrent density, and exhibited prolonged lifetimes of formed charge carriers. Additionally, the studies showed that reuse of the obtained heterostructures is possible without a significant loss of their activity. The hydrothermal method of obtaining the LDH/GCN heterostructure proposed in this study takes place at lower temperatures than usually reported in the literature while maintaining high photoactivity of the material. This work highlights the importance of proper synthesis parameters to obtain heterostructures with a superior photocatalytic activity. The conducted research therefore explored a new insight regarding a cost- and energy-friendly method of obtaining a GCN/LDH photocatalyst that holds significant promise for practical applications in wastewater treatment. Future work will focus on further improvement of hydrothermal synthesis conditions as well as scaling this technology to evaluate its efficacy in dynamic flow-through reactors.

## Supporting Information Available

Additional experimental details and results regarding materials’ characterization and photocatalytic activity (PDF)
