# Supplementary material for: Tuning the Structural and Electronic Properties of Zn–Cr LDH/GCN Heterostructure for Enhanced Photodegradation of Estrone in UV and Visible Light
Source: Langmuir. 2024 Aug 14;40(34):18163–75. doi: 10.1021/acs.langmuir.4c01897 (PMC11363147; doi:10.1021/acs.langmuir.4c01897)
Supplement: Supplementary file 1 — la4c01897_si_001.pdf [file la4c01897_si_001.pdf]

Supporting Information

Tuning the structural and electronic properties of Zn-Cr LDH/GCN heterostructure for enhanced photodegradation of estrone in UV and visible light

Anna Jędras[1], Jakub Matusik[1], Esakkinaveen Dhanaraman[2], Yen-Pei Fu[2], Grzegorz Cempura[3]*

[1] AGH University of Krakow; Faculty of Geology, Geophysics and Environmental Protection; Department of Mineralogy, Petrography and Geochemistry, al. Mickiewicza 30, 30-059 Krakow, Poland.

[2] National Dong Hwa University, Department of Materials Science and Engineering, Shou-Feng, Hualien 97401, Taiwan

[3] AGH University of Krakow, Faculty of Metal Engineering and Industrial Computer Science, International Centre of Electron Microscopy for Materials Science, al. Mickiewicza 30, 30-059 Krakow, Poland.

Corresponding Author: A. Jędras (ajedras@agh.edu.pl)

Table of contents:

HPLC measurements conditions 3

Figure S1. XRD patterns of all synthesized materials: (a) heterostructures obtained through coprecipitation, (b) heterostructures obtained through adsorption-coprecipitation, (c) hydrothermal heterostructures. 3

Table S1. FWHM values calculated for the LDH 11.50° peak with the use of the Pseudo-Voigt function fitting in the Profex software. 4

Table S2. Specific surface area (S_{BET}) values for all obtained materials. 4

Figure S2. FTIR spectra of all obtained materials: (a) heterostructures obtained through coprecipitation, (b) heterostructures obtained through adsorption-coprecipitation, (c) hydrothermal heterostructures. 5

Figure S3. BF&HAADF-STEM images of the: (a) LDH, (b) GCN, (c) HLDH, (d) CHLDH-G50, (e) ACLDH-G50, (f) HLDH-G50. 6

Figure S4. XPS spectra of (a) N 1s, (b) C 1s, (c) Zn 2p, (d) Cr 2p, (e) O 1s for the GCN, LDH, HLDH, CLDH-G50, ACLDH-G50, and HLDH-G50 materials. 7

Table S3. Zn/Cr and C/N ratios, based on XRF and CHN measurements, respectively................ 8

Figure S5. Tauc plots for the (a) GCN, (b) LDH, (c) HLDH, and VB-XPS of (d) GCN, (e) LDH, (f) HLDH. 9

Table S4. Emission Decay Components for the LDH, HLDH, GCN, and heterostructures with 50% GCN content. 10

Figure S6. Photocatalytic degradation of estrone (a-c) in UV and (d-f) visible light for all of the obtained materials. (g-i) Adsorption kinetics performed in the dark for all of the obtained materials. 10

Table S5. C/C_0 (-) values with standard deviations of photocatalytic and adsorption experiments for the GCN, LDH, HLDH, CLDH-G50, ACLDH-G50, and HLDH-G50. 11

HPLC measurements conditions

Chromatographic separation was carried out on an ARION Polar C18 HPLC column (3.0 µm, 150 mm x 4.6 mm) protected by an ARION guard system (3.0 µm, 5 mm cartridges). The temperature set in the oven was 40ºC. The mobile phases used were acetonitrile and water in a 60:40 volume ratio, respectively. The flow rate was equal to 1.0 mL/min and the injection volume was 100 µL. The UV-Vis detector wavelength was set to 280 nm. The retention time for estrone in these conditions was 4.8 min.

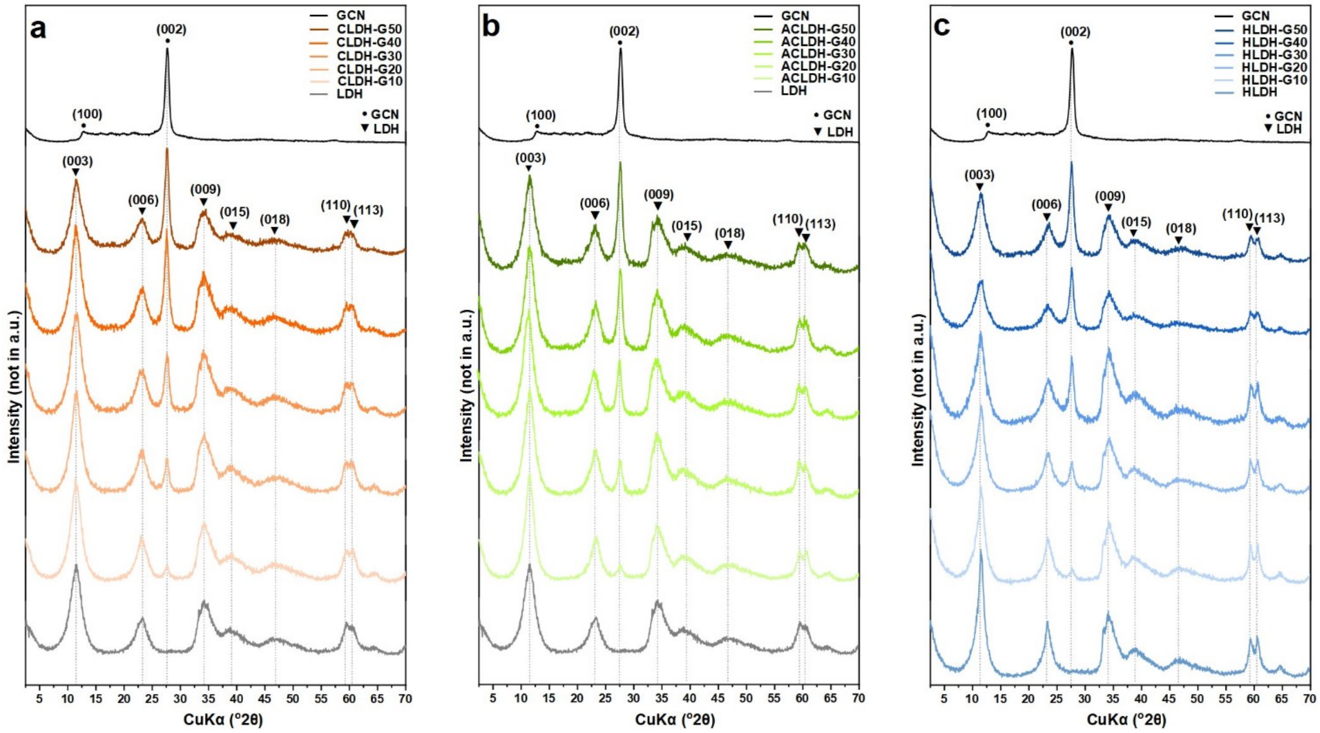

Figure S1. XRD patterns of all synthesized materials: (a) heterostructures obtained through coprecipitation, (b) heterostructures obtained through adsorption-coprecipitation, (c) hydrothermal heterostructures.

Table S1. FWHM values calculated for the LDH 11.50° peak with the use of the Pseudo-Voigt function fitting in the Profex software.

Material	FWHM (°)
CLDH-G10	2.17
CLDH-G20	2.20
CLDH-G30	2.35
CLDH-G40	2.34
CLDH-G50	2.37
ACLDH-G10	1.79
ACLDH-G20	2.17
ACLDH-G30	2.19
ACLDH-G40	2.23
ACLDH-G50	2.33
HLDH-G10	1.58
HLDH-G20	1.61
HLDH-G30	1.99
HLDH-G40	2.22
HLDH-G50	2.35
LDH	2.48
HLDH	1.24

Table S2. Specific surface area (S_{BET}) values for all obtained materials.

Material	S_{BET} (m^2/g)
CLDH-G10	71.7
CLDH-G20	87.7
CLDH-G30	70.0
CLDH-G40	102.8
CLDH-G50	95.9
ACLDH-G10	72.7
ACLDH-G20	89.9
ACLDH-G30	104.0
ACLDH-G40	101.5
ACLDH-G50	107.6
HLDH-G10	129.9
HLDH-G20	172.3
HLDH-G30	114.9
HLDH-G40	124.6
HLDH-G50	124.0
LDH	91.6
HLDH	192.2
GCN	13.0

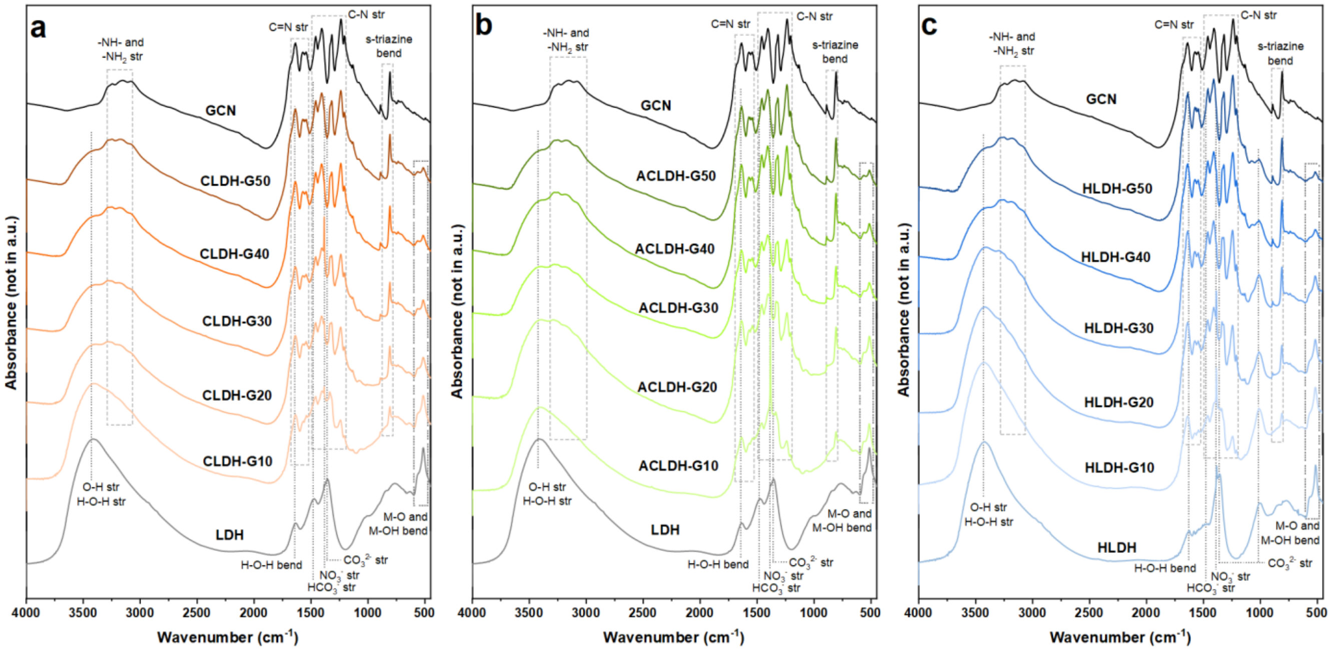

Figure S2. FTIR spectra of all obtained materials: (a) heterostructures obtained through coprecipitation, (b) heterostructures obtained through adsorption-coprecipitation, (c) hydrothermal heterostructures.

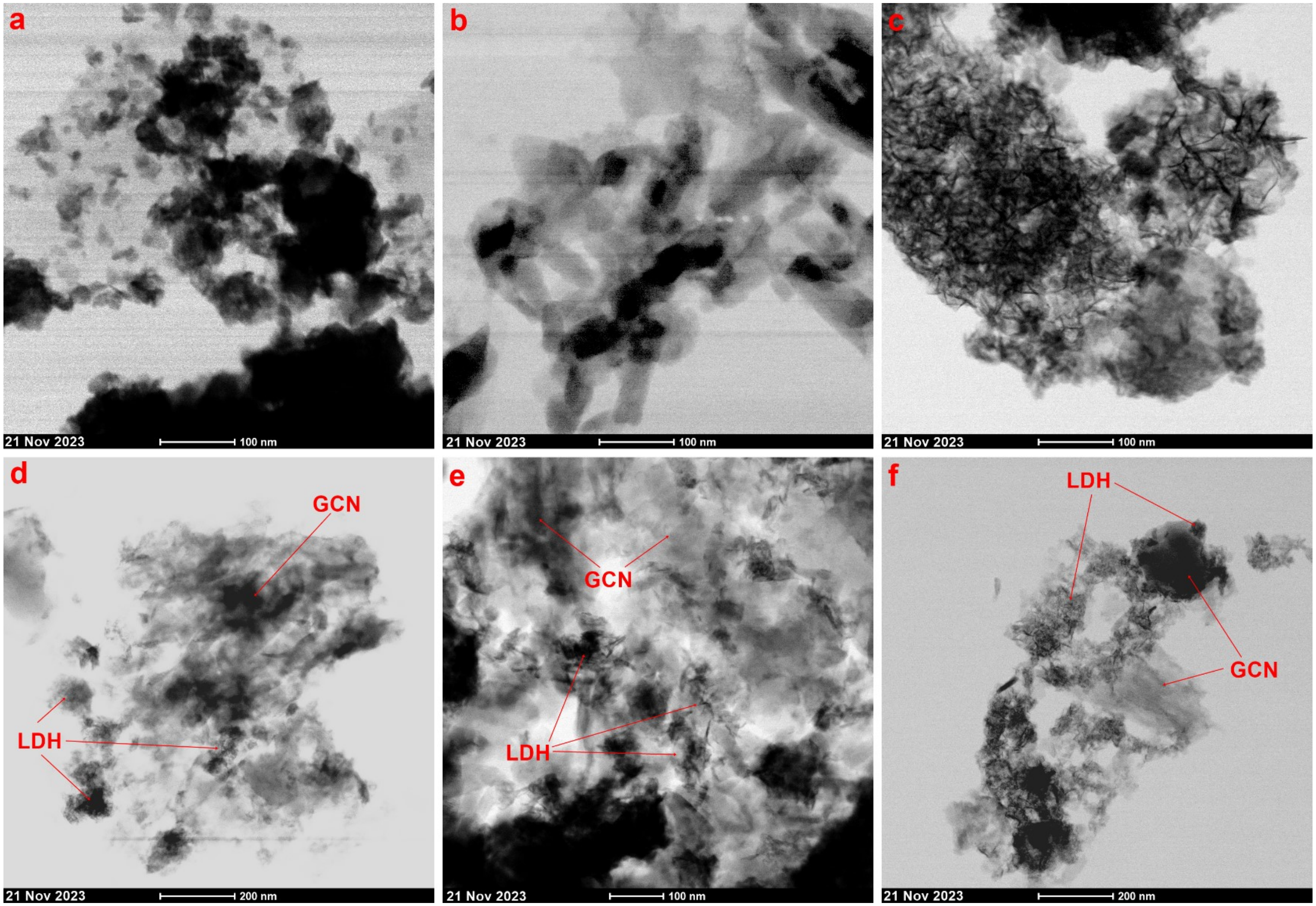

Figure S3. BF&HAADF-STEM images of the: (a) LDH, (b) GCN, (c) HLDH, (d) CHLDH-G50, (e) ACLDH-G50, (f) HLDH-G50.

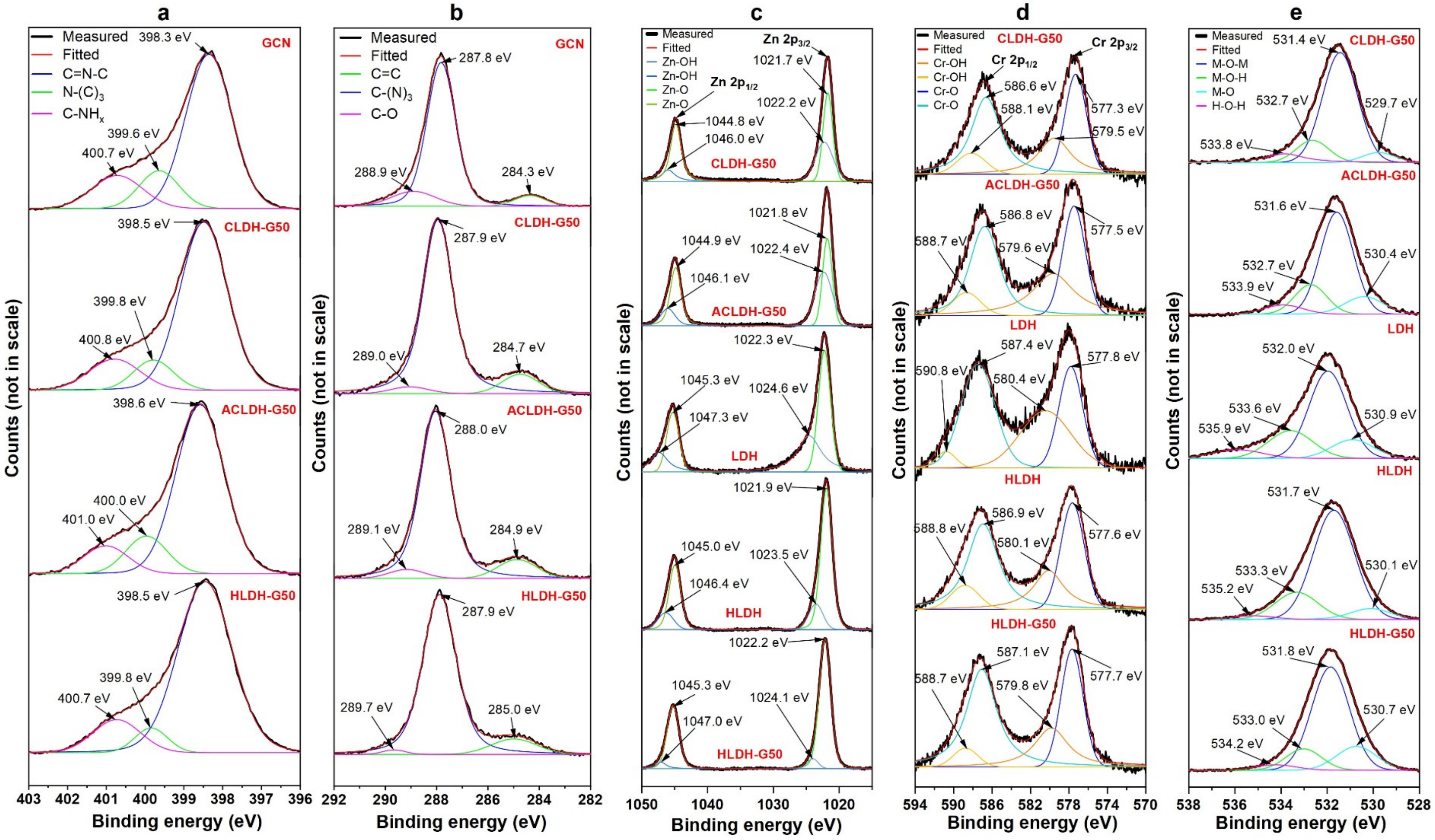

Figure S4. XPS spectra of (a) N 1s, (b) C 1s, (c) Zn 2p, (d) Cr 2p, (e) O 1s for the GCN, LDH, HLDH, CLDH-G50, ACLDH-G50, and HLDH-G50 materials.

Table S3. Zn/Cr and C/N ratios, based on XRF and CHN measurements, respectively.

Material	XRF			CHN			
	**% ZnO**	**% Cr_2O_3**	**Zn/Cr ratio**	**C (wt. %)**	**N (wt. %)**	**N/C ratio**	**GCN (wt. %)**
CLDH-G10	19.83	11.47	2.03	7.14	5.21	0.73	7.69
CLDH-G20	25.36	14.00	2.13	8.41	11.07	1.32	17.2
CLDH-G30	28.90	15.92	2.13	12.18	19.32	1.59	30.55
CLDH-G40	32.69	18.19	2.11	21.95	22.07	1.01	35.06
CLDH-G50	33.71	18.61	2.13	18.45	31.04	1.68	49.57
ACLDH-G10	20.01	11.43	2.06	5.45	5.75	1.06	8.57
ACLDH-G20	22.04	12.88	2.01	8.70	12.06	1.39	18.79
ACLDH-G30	25.37	14.23	2.09	11.38	17.16	1.51	27.08
ACLDH-G40	35.21	19.36	2.14	14.32	24.08	1.68	38.29
ACLDH-G50	36.84	18.75	2.31	17.42	29.99	1.72	47.88
HLDH-G10	22.46	11.86	2.22	5.48	4.75	0.87	6.1
HLDH-G20	22.94	11.66	2.31	7.27	10.55	1.45	15.61
HLDH-G30	30.77	15.91	2.27	9.69	16.49	1.70	25.34
HLDH-G40	34.01	17.85	2.24	14.10	24.27	1.72	38.04
HLDH-G50	37.26	19.84	2.21	16.49	28.74	1.74	45.4
LDH	41.13	21.66	2.23	7.60	0.48	0.06	0.00
HLDH	35.82	17.30	2.43	12.61	1.06	0.08	0.00
GCN	-	-	-	34.74	62.13	1.79	100.00

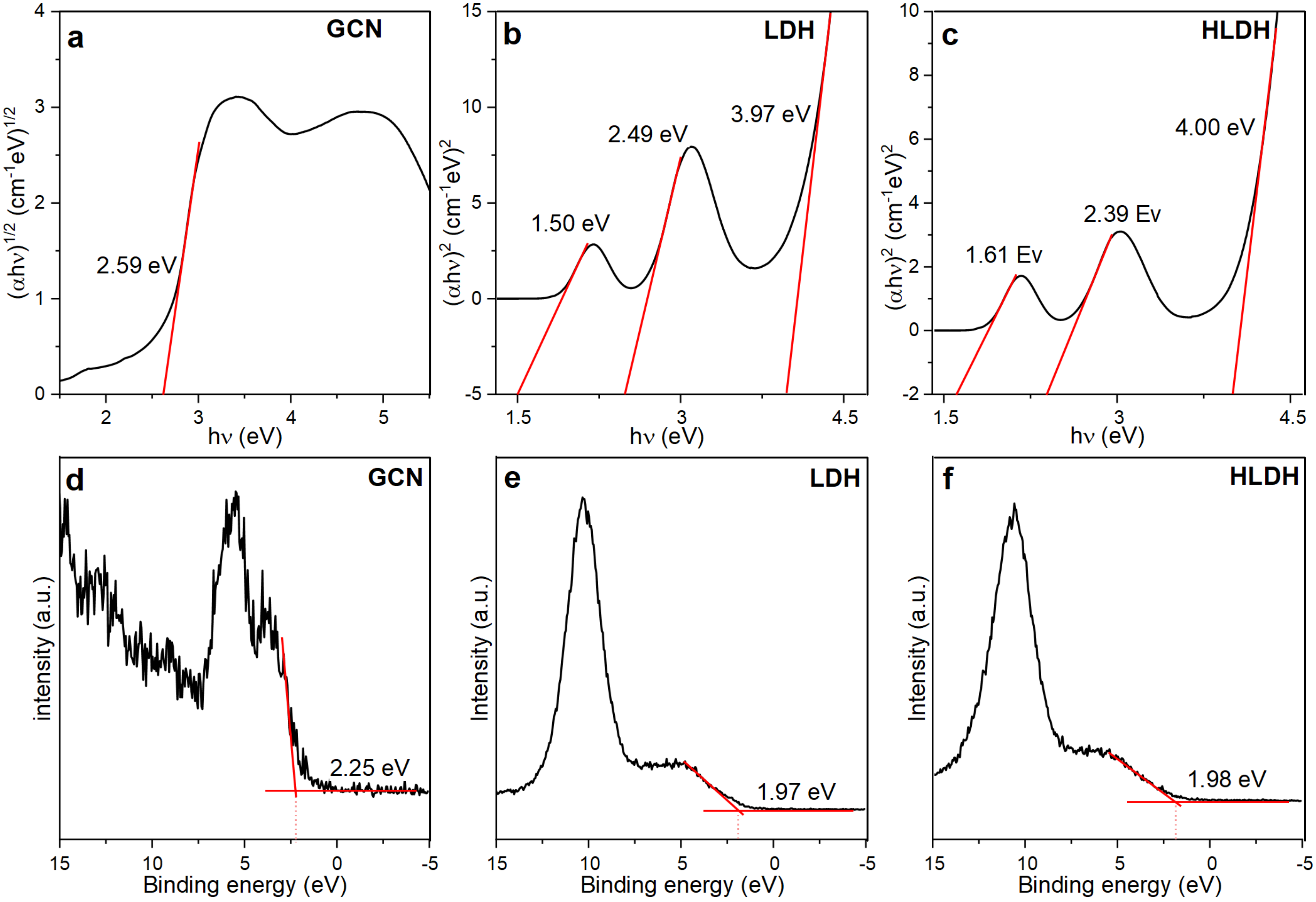

Figure S5. Tauc plots for the (a) GCN, (b) LDH, (c) HLDH, and VB-XPS of (d) GCN, (e) LDH, (f) HLDH.

Table S4. Emission Decay Components for the LDH, HLDH, GCN, and heterostructures with 50% GCN content.

		LDH	SD	HLDH	SD	GCN	SD	CLDH-G50	SD	ACLDH-G50	SD	HLDH-G50	SD
Decay time (ns)	τ_1	1.52	0.01	1.54	0.01	2.54	0.01	2.51	0.01	2.98	0.01	2.94	0.01
	τ_2	-	-	-	-	16.80	0.37	20.15	1.01	22.00	0.75	23.47	0.93
	τ_{av}	1.52	0.01	1.54	0.01	2.64	0.01	2.52	0.01	3.00	0.02	2.96	0.02
Fractional contribution	A_1	2897.41	111.89	2204.06	84.75	132.42	2.60	135.46	3.22	69.97	1.29	76.10	1.36
	A_2	-	-	-	-	0.15	0.01	0.07	0.01	0.09	0.01	0.07	0.00

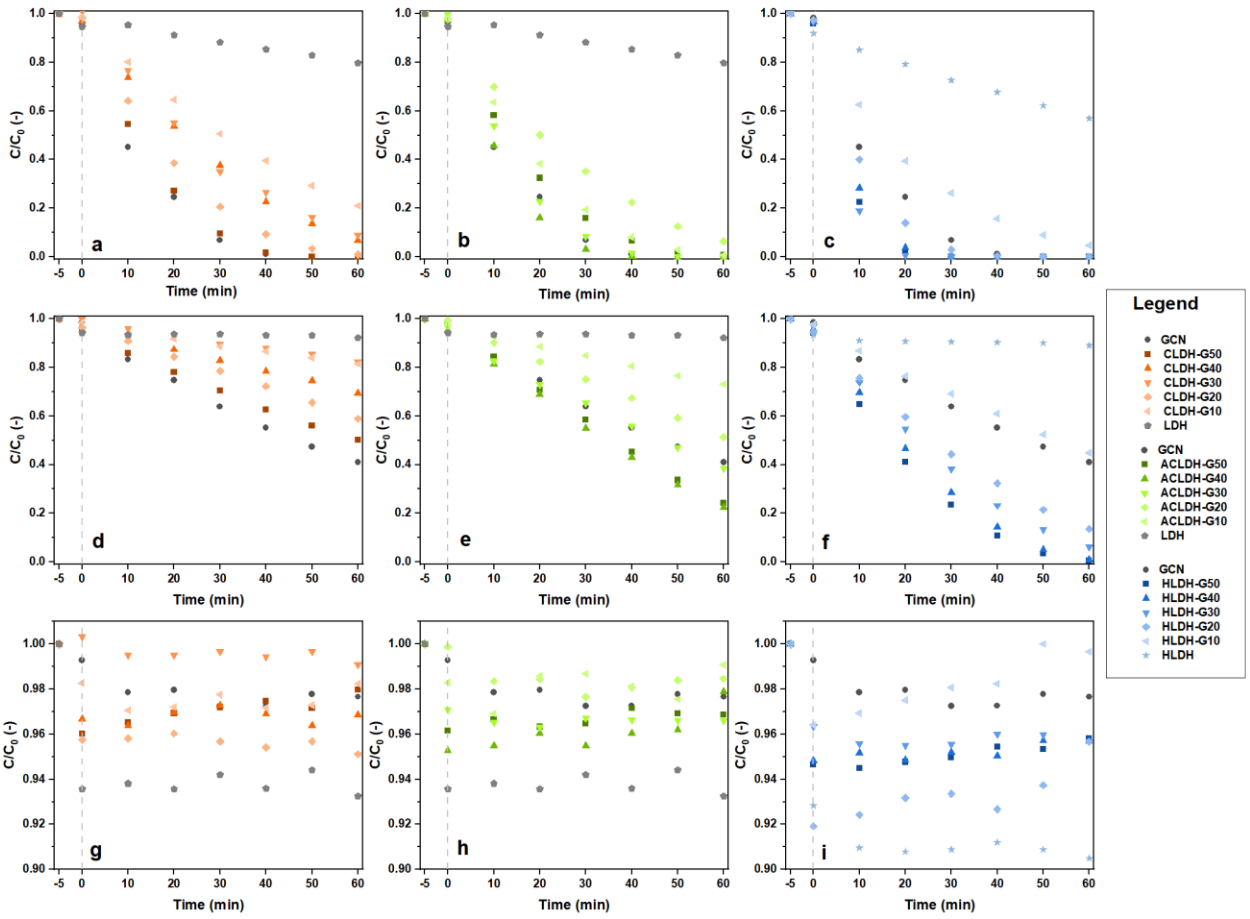

Figure S6. Photocatalytic degradation of estrone (a-c) in UV and (d-f) visible light for all of the obtained materials. (g-i) Adsorption kinetics performed in the dark for all of the obtained materials.

Table S5. C/C_0 (-) values with standard deviations of photocatalytic and adsorption experiments for the GCN, LDH, HLDH, CLDH-G50, ACLDH-G50, and HLDH-G50.

UV light photocatalysis

Material \ Time (min)	-5	0	SD	10	SD	20	SD	30	SD	40	SD	50	SD	60	SD
GCN	1	0.9820	0.0017	0.4511	0.0005	0.2454	0.0009	0.0682	0.0004	0.0101	0.0004	b.d.	-	b.d.	-
LDH	1	0.9463	0.0027	0.9538	0.0170	0.9114	0.0151	0.8817	0.0137	0.8538	0.0089	0.8288	0.0093	0.7976	0.0046
HLDH	1	0.9196	0.0017	0.8510	0.0037	0.7915	0.0026	0.7258	0.0008	0.6767	0.0003	0.6222	0.0014	0.5694	0.0014
CLDH-G50	1	0.9513	0.0044	0.5454	0.0197	0.2710	0.0094	0.0941	0.0035	0.0168	0.0013	b.d.	-	b.d.	-
ACLDH-G50	1	0.9693	0.0009	0.5831	0.0008	0.3240	0.0012	0.1577	0.0015	0.0659	0.0005	0.0196	0.0006	0.0065	0.0005
HLDH-G50	1	0.9584	0.0001	0.2255	0.0008	0.0193	0.0001	b.d.	-	b.d.		b.d.	-	b.d.	-

Visible light photocatalysis

Material \ Time (min)	-5	0	SD	10	SD	20	SD	30	SD	40	SD	50	SD	60	SD
GCN	1	0.9854	0.0010	0.8339	0.0031	0.7468	0.0017	0.6382	0.0018	0.5522	0.0022	0.4734	0.0021	0.4092	0.0017
LDH	1	0.9427	0.0197	0.9347	0.0182	0.9369	0.0171	0.9368	0.0190	0.9330	0.0172	0.9314	0.0191	0.9213	0.0171
HLDH	1	0.934	0.0036	0.9101	0.0038	0.9071	0.0024	0.9050	0.0004	0.9027	0.0019	0.9010	0.0014	0.8897	0.0011
CLDH-G50	1	0.9592	0.0027	0.8587	0.0051	0.7801	0.0045	0.7038	0.0032	0.6269	0.0031	0.5597	0.0023	0.5007	0.0020
ACLDH-G50	1	0.9853	0.0013	0.8442	0.0036	0.7082	0.0004	0.5844	0.0024	0.4529	0.0012	0.3365	0.0003	0.2424	0.0005
HLDH-G50	1	0.9412	0.0002	0.6476	0.0005	0.4107	0.0001	0.2340	0.0001	0.1081	0.0005	0.0347	0.0001	0.0053	0.0003

Adsorption (no light)

Material \ Time (min)	-5	0	SD	10	SD	20	SD	30	SD	40	SD	50	SD	60	SD
GCN	1	0.9929	0.0051	0.9786	0.0021	0.9796	0.0044	0.9726	0.0029	0.9727	0.0036	0.9778	0.0006	0.9765	0.0019
LDH	1	0.9357	0.0022	0.9382	0.0026	0.9356	0.0017	0.9420	0.0019	0.9359	0.0008	0.9441	0.0006	0.9325	0.0010
HLDH	1	0.9284	0.0000	0.9096	0.0007	0.9079	0.0037	0.9089	0.0011	0.9119	0.0014	0.9088	0.0008	0.9050	0.0011
CLDH-G50	1	0.9602	0.0011	0.9654	0.0048	0.9693	0.0021	0.9720	0.0002	0.9746	0.0029	0.9715	0.0003	0.9799	0.0020
ACLDH-G50	1	0.9617	0.0017	0.9667	0.0022	0.9633	0.0027	0.9646	0.0036	0.9715	0.0033	0.9693	0.0035	0.9688	0.0037
HLDH-G50	1	0.9465	0.0041	0.9449	0.0008	0.9477	0.0002	0.9496	0.0003	0.9543	0.0017	0.9534	0.0001	0.9580	0.0005
